# Effects of Al substitution by Si in Ti_3_AlC_2_ nanolaminate

**DOI:** 10.1038/s41598-021-81346-w

**Published:** 2021-02-09

**Authors:** M. A. Hadi, Md Roknuzzaman, M. T. Nasir, U. Monira, S. H. Naqib, A. Chroneos, A. K. M. A. Islam, Jose A. Alarco, Kostya (Ken) Ostrikov

**Affiliations:** 1 Department of Physics, University of Rajshahi, Rajshahi, 6205 Bangladesh; 2 School of Chemistry and Physics and Centre for Materials Science, Queensland University of Technology (QUT), Brisbane, QLD 4000 Australia; 3 School of Physics, University of New South Wales (UNSW Sydney), Kensington, Sydney, NSW 2052 Australia; 4 Department of Physics, Jashore University of Science and Technology, Jashore, 7408 Bangladesh; 5 Department of Arts and Sciences, Bangladesh Army University of Science and Technology, Saidpur, Nilphamari, 5310 Bangladesh; 6 Faculty of Engineering, Environment and Computing, Coventry University, Priory Street, Coventry, CV1 5FB UK; 7 Department of Materials, Imperial College, London, SW7 2AZ UK; 8 International Islamic University Chittagong, Kumira, Chittagong, 4318 Bangladesh

**Keywords:** Materials science, Optics and photonics, Physics

## Abstract

Recently, a series of high-purity Ti_3_(Al_1−*x*_Si_*x*_)C_2_ solid solutions with new compositions (*x* = 0.0, 0.2, 0.4, 0.6, 0.8 and 1.0) have been reported with interesting mechanical properties. Here, we have employed density functional theory for Ti_3_(Al_1−*x*_Si_*x*_)C_2_ solid solutions to calculate a wider range of physical properties including structural, electronic, mechanical, thermal and optical. With the increase of *x*, a decrease of cell parameters is observed. All elastic constants and moduli increase with *x*. The Fermi level gradually increases, moving towards and past the upper bound of the pseudogap, when the value of *x* goes from zero to unity, indicating that the structural stability reduces gradually when the amount of Si increases within the Ti_3_(Al_1−*x*_Si_*x*_)C_2_ system. In view of Cauchy pressure, Pugh’s ratio and Poisson’s ratio all compositions of Ti_3_(Al_1−*x*_Si_*x*_)C_2_ are brittle in nature. Comparatively, low Debye temperature, lattice thermal conductivity and minimum thermal conductivity of Ti_3_AlC_2_ favor it to be a thermal barrier coating material. High melting temperatures implies that the solid solutions Ti_3_(Al_1−*x*_Si_*x*_)C_2_ may have potential applications in harsh environments. In the visible region (1.8–3.1 eV), the minimum reflectivity of all compositions for both polarizations is above 45%, which makes them potential coating materials for solar heating reduction.

## Introduction

Ti_3_AlC_2_ belongs to the family of M_*n*+1_AX_*n*_ (MAX) phases with more than 80 members. These are layered, machinable, nanolaminated ternary carbides, nitrides and borides, where M is an early transition metal, A is an A-group element, mainly from groups 13–16 and X is C or N or B^1–3^. These compounds possess hexagonal crystal structure with space group *P*6_*3*_/*mmc* (space group number: 194), where M atoms are in a near closed packed arrangement and are intercalated with A-group atomic layers, with the X-atoms residing in the octahedral sites between the M layers. These atomic arrangements give the MAX phases an effective laminated layered structure that leads to the naming of the MAX phase as nanolaminates^4^. MAX phases possess a unique combination of properties, both metallic and ceramic in nature due to the layered structure. Metallic properties can include good thermal and electrical conductivities, good thermal shock resistance, excellent damage tolerance, and good machinability. Ceramic properties include low density, elastic rigidity, oxidation and creep resistance, and the ability to maintain the strength up to very high temperatures^3–5^.

These properties constitute MAX phases technologically important materials. There are potential uses of MAX phases as high temperature heating elements, tough, machinable and thermal shock resistant refractories, neutron irradiation resistant parts for nuclear applications, coatings for electrical contacts, precursor for the synthesis of carbide-derived carbon and MXenes, a family of two-dimensional transition metal carbides, nitrides, borides, and carbonitrides^3–6^.

The structures as well as the properties of MAX phases can be altered, and even improved, by hosting a new element into the lattice. The inclusion of a new element on the M, A, and/or X sites leads to isostructural MAX phase solid solutions. In recent time, a growing interest in MAX phase solid solutions is seen in the scientific community as a way to obtain improved properties. For example the phase Ti_3_SiC_2_ is found to be more oxidation resistant when Al on A-site or Nb on M-site is introduced to form Ti_3_(Si_1−*x*_Al_*x*_)C_2_ or (Nb_1−*x*_Ti_*x*_)_3_SiC_2_ solid solutions^7,8^. Meng et al*.*^9^ and Barsoum et al*.*^10^ reported on the solid solution hardening effect for (V_*x*_Ti_1−*x*_)_2_AlC and Ti_2_AlC_*x*_N_*y*_, respectively. A solid solution softening effect was reported by Bei et al*.*^11^ for Ti_3_(Al_1−*x*_Sn_*x*_)C_2_. The partial inclusion of Ti on the M-site in Nb_4_AlC_3_ improves the mechanical properties^12^, flexural strength, fracture toughness and Vickers hardness^13^ significantly. Structural, magnetic, and electrical and thermal transport properties are observed to modify as the *x*-content in Cr_2−*x*_M_*x*_GeC (M = Ti, V, Mn, Fe, and Mo) increases^14^. M-site solid solutions with partial inclusion of Zr, Hf, and Nb enhance the elastic moduli and strength of Ti_3_SiC_2_ at elevated temperatures^15^. Incorporation of Si in Ti_3_AlC_2_ can improve Young’s and shear moduli significantly^16^. According to a theoretical study, the partial inclusion of Nb atoms in Ti_2_SC and Zr_2_SC improves the mechanical strength^17^.

M-site solid solutions (Zr_3−*x*_Ti_*x*_)AlC_2_ have been reported recently and a density functional theory (DFT) calculation described their mechanical and thermodynamic properties^18,19^. Synthesis of Zr_3_(Al_1−*x*_Si_*x*_)C_2_ has been associated with an improvement of the free energy barrier for nucleation, without preferential, competitive phase formation^20^. Successful synthesis of (Zr,M)_2_AlC and Zr_2_(Al,A)C compounds with variable compositions has been reported^21,22^. Furthermore, alloying effects on the M-site in M_2_AlC (M = Ti, V, Zr, and Hf) with elements in the first transition metal row as well as Ca and Sc have been investigated theoretically^23^. Meanwhile, phase stability and physical properties of (Zr_1−*x*_Nb_*x*_)_2_AlC MAX phases have also been predicted recently^24^.

A renewed interest on MAX phases has grown in the scientific community after the synthesis of phase pure, dense bulk Ti_3_SiC_2_ in 1996^25^. This phase has become among the most studied MAX phases due to the combination of strength, machinability and ductility at high temperatures, in addition to non-susceptibility to thermal shock, never observed previously in any other MAX phase material^26^. On the other hand, Ti_3_AlC_2_ is the only ceramic that exhibits room temperature compressive plasticity^27,28^. Moreover, Ti_3_AlC_2_ exhibits excellent oxidation resistance at high temperature due to formation of a pure alumina protective oxide layer^29^. For this reason, it is expected that the MAX phase solid solutions Ti_3_(Al_1−*x*_Si_*x*_)C_2_ may be enriched with a good set of properties to make them technologically important materials. It is thus not surprising that these solid solutions have been investigated extensively both experimentally and theoretically^7,30–38^. All previous theoretical and experimental studies on Ti_3_(Al_1−*x*_Si_*x*_)C_2_ have been accomplished with *x* = 0, 0.25, 0.33, 0.37, 0.50, 0.625, 0.67, 0.75, 0.875, and 1.0. The samples prepared in previous experimental studies were not highly pure. So, the physical properties described in those studies were not able to address properly the Ti_3_(Al_1−*x*_Si_*x*_)C_2_ solid solutions. In the same manner, the theoretical results cannot be justified accurately.

Recently, a series of new high-purity Ti_3_(Al_1−*x*_Si_*x*_)C_2_ solid solutions with varying compositions (*x* = 0.0, 0.2, 0.4, 0.6, 0.8 and 1.0) have been reported and their lattice parameters, elastic modulus, heat capacities and thermal expansion coefficient have been experimentally investigated to examine the effects of the amount of Si in Ti_3_(Al_1−*x*_Si_*x*_)C_2_ solid solutions on their structural, physical and mechanical properties^16^. The present DFT study aims to investigate the physical properties of Ti_3_(Al_1-−_Si_*x*_)C_2_ solid solutions from a theoretical perspective and to validate the DFT based first-principles methods using the more recently available experimental results.

## Results and discussions

### Structural properties

**Figure 1:**
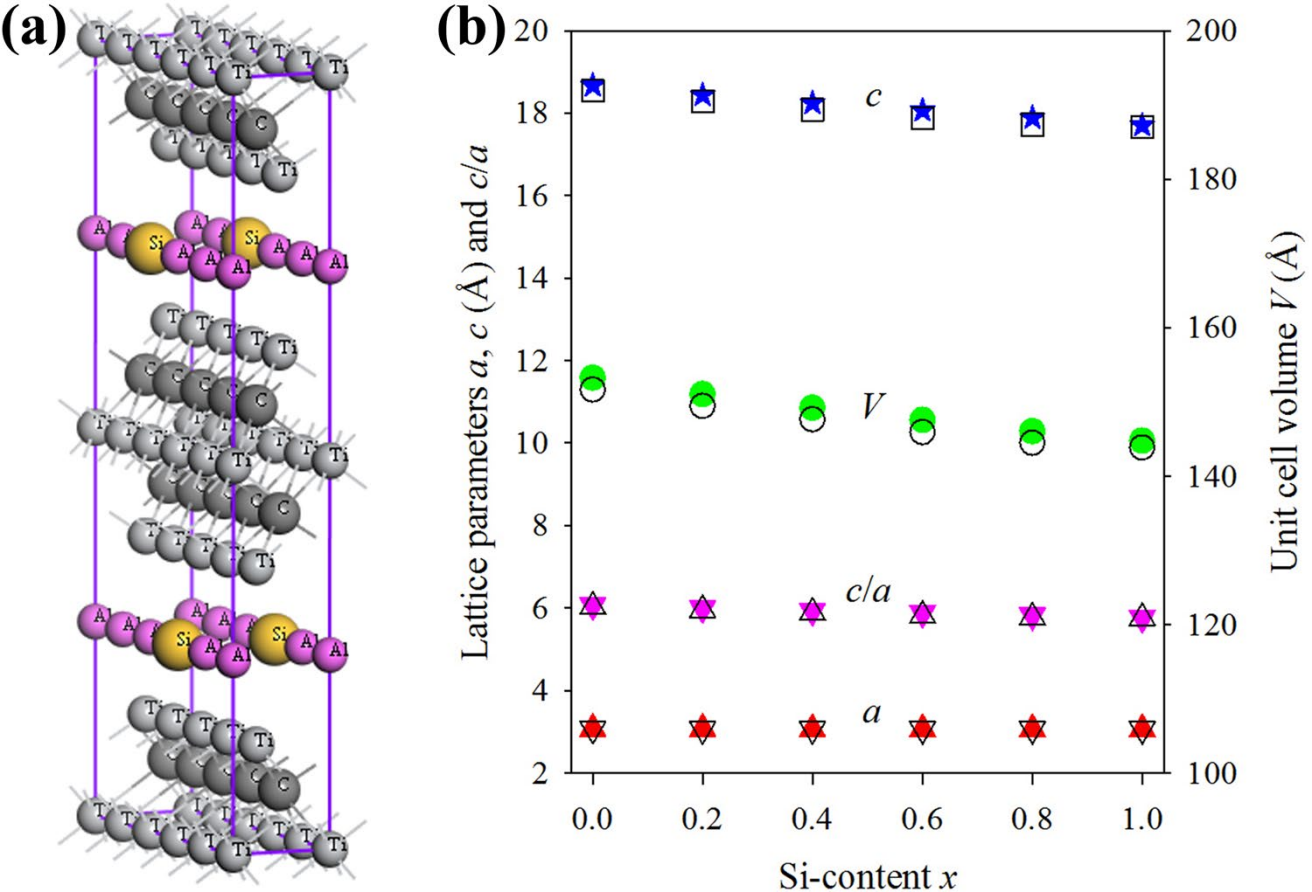
Structural properties for the considered MAX solid solutions. (**a**) Supercell of Ti_3_(Al_1−*x*_Si_*x*_)C_2_ with *x* = 0.2. (**b**) Structural properties of Ti_3_(Al_1−*x*_Si_*x*_)C_2_ as a function of Si-content *x*. Solid and open symbols represent the calculated and experimental^16^ values, respectively.

The supercell of Ti_3_(Al_1−*x*_Si_*x*_)C_2_ with *x* = 0.2 is shown in Fig. 1a. The optimized lattice parameters are listed in Supplementary Table 1 along with the experimental values. Figure 1b also shows these results as a function of Si content *x*, where the near linear trend indicates a good agreement with Vegard’s law for solid solutions^39^. With the increase of silicon content *x*, a decrease of cell parameters (negligible in *a* and significant in *c* as well as in *V*) is observed. The Al-containing Ti_3_AlC_2_ has a unit cell larger than that of the Si-containing Ti_3_SiC_2_ as the Al has larger covalent atomic radius (1.18 Å) compared to that of Si (1.11 Å). The deviation of lattice parameters calculated in this study from the corresponding experimental values is within 0.2% for *a*, 1.1% for *c* and 1.2% for *V*, indicating the reliability of the present investigations. The GGA trend in lattice parameters is also observed in this study.

**Table 1:** Single crystal elastic constants *C*_ij_, elastic moduli (*B*, *G*, *E*) and Cauchy pressure *P*_c_ = *C*_12_–*C*_44_ (in GPa), Pugh’s ratio *B*/*G* and Poisson’s ratio *v* of Ti_3_(Al_1−*x*_Si_*x*_)C_2_.

*x*	*C*_11_	*C*_33_	*C*_44_	*C*_12_	*C*_13_	*B*	*G*	*E*	*B*/*G*	*v*	*P*_c_
0.0	356	293	122	73	67	157	130	305	1.210	0.176	− 49
0.2	360	307	131	76	73	163	134	316	1.215	0.177	− 55
0.4	364	324	141	77	80	170	139	328	1.219	0.178	− 64
0.6	365	335	144	83	85	174	140	331	1.245	0.183	− 61
0.8	364	344	152	84	93	179	142	337	1.262	0.186	− 68
1.0	368	354	159	85	98	184	145	345	1.265	0.187	− 74

### Electronic and bonding properties

The ground state electronic band structures of Ti_3_(Al_1−*x*_Si_*x*_)C_2_ calculated along high symmetry directions in the first Brillouin zone are shown in Supplementary Fig. 1. The band structures show dense regions of valence and conduction bands connected by highly dispersive bands within a window of about 2 eV around the Fermi level, indicating metallic bonding nature of all compositions of Ti_3_(Al_1−*x*_Si_*x*_)C_2_. The overlap of the dispersive bands and the dense conduction band region occurs at the Γ-point at about 1 eV above the Fermi level. This intersection (or overlap) point shows the tendency to be closer to the Fermi level as Si-content *x* increases from 0 to 1. The energy dispersions along different directions are different, indicating the lattice anisotropy in Ti_3_(Al_1−*x*_Si_*x*_)C_2_. The lowest valence bands occupy the energy states between –12 and –9.6 eV. A band gap above the lowest valence bands appears for *x* = 0. This gap reduces gradually as *x* increases and fully disappears for *x* = 0.6 and an intermediate valence band appears between the upper and lower valence bands (see DOS profiles). The increase of Si-content *x* significantly reduces the d-resonance around the Fermi level of the transition metal Ti. This can be seen more clearly in Supplementary Fig. 2, wherein the electronic density of states (DOS) is depicted.

**Figure 2:**
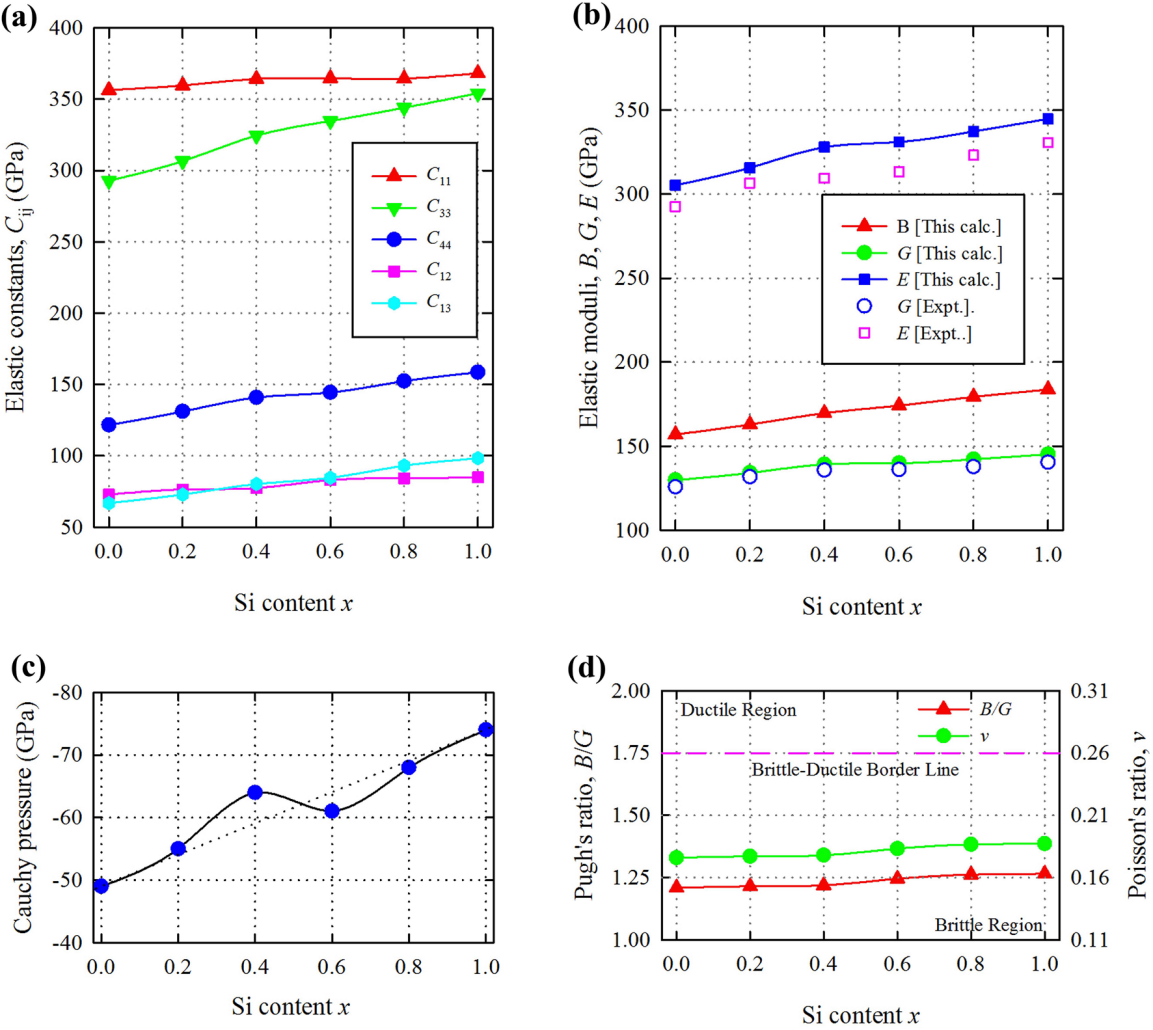
Variation of the investigated mechanical properties as a function of Si-content *x* in Ti_3_(Al_1−*x*_Si_*x*_)C_2_ solid. (**a**) Elastic constants. (**b**) Elastic moduli. (**c**) Cauchy pressure. (**d**) Pugh’s and Poisson’s ratio.

In order to understand the nature of chemical bonding in Ti_3_(Al_1−*x*_Si_*x*_)C_2_, the total density of states (TDOS) as well as the partial density of states (PDOS) are calculated and shown in Supplementary Fig. 2. The Fermi level is denoted with a vertical broken line, which is set at 0 eV in the energy scale defined by *E* − *E*_F_. For *x* = 0, i.e., for Ti_3_AlC_2_, the TDOS in the Fermi level is placed at a dip, which is known as a pseudogap, signifying the structural stability of this phase. The Fermi level gradually moves away from the pseudogap towards the upper bound (right direction) when the value of the Si content *x* goes from zero to unity. This means that the structural stability reduces gradually when the amount of Si increases within the Ti_3_(Al_1−*x*_Si_*x*_)C_2_ system. The finite value *N*(*E*_F_) of TDOS at the Fermi level for all *x* values resulting from d-resonance of the transition metal Ti indicates metallic bonding of Ti_3_(Al_1−*x*_Si_*x*_)C_2_. *N*(*E*_F_) decreases gradually up to *x* = 0.6 and then increases but the value of *N*(*E*_F_) for *x* = 1.0 does not exceed the value for *x* = 0.0.

The lowest valence band situated between –12 and –9.2 eV arises due to hybridization of C 2s with Ti 3d electrons in the two end members of Ti_3_(Al_1−*x*_Si_*x*_)C_2_. In other cases, together with C 2s orbital, almost equal contribution comes from Si 3s electrons. All of these interactions indicate covalent Ti-C and Ti-Si bonding. The higher valence band consists of several distinct peaks. The left peak of the middle region (–4 to –1 eV) arises as a result of interaction between C 2p and Ti 3d electrons for two end members of Ti_3_(Al_1−*x*_Si_*x*_)C_2_. For *x* = 0.2 and 0.4, mainly Si 3p and C 2p lead to form such peaks, while for *x* = 0.6 and 0.8, Al 2p instead of Si 3p contributes similarly. For the two end members, the middle maximum is due to C 2p and Ti 3d electrons. For *x* = 0.2 and 0.4, an additional large contribution comes from Si 3p electrons, while for *x* = 0.6 and 0.8, this contribution comes from Al 2p electrons. The right peak of the middle region is close to the Fermi level, which corresponds to the hybridization between Ti 3d and Al 2p/Si 3p electrons in two end members, respectively. This hybridization leads to weaker Ti–Al and Ti-Si covalent bonding in Ti_3_AlC_2_ and Ti_3_SiC_2_, respectively. The bond length between Ti and Al (2.908 Å) is larger than that of between Ti and Si (2.701 Å) when Al is entirely substituted by Si. On the other hand, the bond population of Ti–Al bond (0.60) is smaller than that of Ti-Si bond (0.76) when Al is completely replaced by Si. Therefore, the Ti-Si bond is stronger than the Ti–Al bond. The relative strengths and lengths of these particular bonds are responsible for the increase of elastic constants and moduli and hence the mechanical strength of Ti_3_(Al_1−*x*_Si_*x*_)C_2_ with the increase of *x* (refer to Table 1). For *x* = 0.2–0.8, the right peak indicates the presence of both Ti–Al and Ti-Si covalent bonding. The conduction band for all *x* arises mainly from the Ti 3d electrons. For pure solid solutions (*x* = 0.2–0.8), the contributions in chemical bonding are different from those of the two end members. It is expected that this difference will lead to significant modification of the physical properties of the two end members. The chemical bonding in the solid solutions Ti_3_(Al_1−*x*_Si_*x*_)C_2_ is obviously a mixture of metallic, covalent and, due to the difference in electronegativity between the comprising elements, ionic in nature.

### Mechanical properties

Knowledge of elastic constants is of high importance to analyze the mechanical behaviors of crystalline solids. MAX phases have five independent elastic constants *C*_ij_, namely, *C*_11_, *C*_33_, *C*_44_, *C*_12_, and *C*_13_ due to their hexagonal crystal symmetry. The calculated elastic constants of Ti_3_(Al_1−*x*_Si_*x*_)C_2_ for Si-content *x* = 0.0, 0.2, 0.4, 0.6, 0.8, and 1.0 are listed in Table 1 and shown in Fig. 2a. All compositions are mechanically stable as they satisfy the Born criteria^40^. A monotonous increase in elastic constants is observed with the increase of Si-content *x*. It means that the mechanical strength and stiffness increase with Si content *x*. *C*_33_ increases at a faster rate and *C*_12_ at a slower rate than other *C*_ij_. This means that the elastic stiffness along the c-axis increases with Si content *x* at a faster rate than that along the a- and b-axes.

Bulk and shear moduli (*B* and *G*) are calculated using the Voigt–Reuss–Hill (VRH) approximation^41^. These parameters are listed in Table 1 and shown in Fig. 2b. Both moduli increase with Si content *x*. The calculated shear modulus *G* is consistent with the measured values^16^ in the whole compositional range. The larger the shear modulus, the more rigid the material behaves. This leads to evaluate the sensitivity of the material to strain variation. The shear modulus assesses the elastic behavior of a material due to shear loading. With Si content *x*, the rigidity of Ti_3_(Al_1−*x*_Si_*x*_)C_2_ increases almost linearly.

The Young’s modulus *E* is calculated from *B* and *G* by using: *E* = 9*BG*/(3*B* + *G*). This modulus is somewhat larger than the measured values at room temperature^16^. The reason is that the higher the temperatures the lower the Young’s modulus. After that their increasing trends with Si content *x* are almost similar. This modulus measures the resistance of a material to elastic deformation under a load. A stiff material has a high Young's modulus and changes its shape only slightly under elastic loads. The Si content *x* increases the stiffness of Ti_3_(Al_1−*x*_Si_*x*_)C_2_. The Young’s modulus is also related to the thermal shock resistance *R*^5^. The lower the Young’s modulus, the higher the thermal shock resistance. Therefore, Ti_3_AlC_2_ has better thermal shock resistance among all compositions of Ti_3_(Al_1−*x*_Si_*x*_)C_2_.

The Cauchy pressure defined as *P*_c_ = *C*_12_ − *C*_44_ is shown in Fig. 2c. It can evaluate the failure modes of solids and the nature of chemical bonding^5^. A negative (positive) value of *P*_c_ is always associated with the brittle (ductile) failure of solids. Accordingly, the solid solution Ti_3_(Al_1−*x*_Si_*x*_)C_2_ is brittle in nature and its brittleness increases monotonically with the Si content *x*. A negative Cauchy pressure corresponds to directional covalent bonding with angular character, while a positive Cauchy pressure signifies metallic bonding. Therefore, all compositions of Ti_3_(Al_1−*x*_Si_*x*_)C_2_ are dominated by directional covalent bonding and its covalency increases with the increase of Si content *x*.

The Pugh’s ratio *B*/*G* serves as a decisive factor for classifying solid materials into brittle and ductile groups. A value of 1.75 for *B*/*G* plays this role and is known as the border line for separating the brittle materials from ductile ones^5^ (see Fig. 2d). Values lower (higher) than this value is associated with a brittle (ductile) material. It is evident that all compositions of Ti_3_(Al_1−*x*_Si_*x*_)C_2_ are brittle in nature as is predicted from the Cauchy pressure.

The Poisson’s ratio *v* is used to predict many physical properties of materials. The stability of a compound against shear can be predicted by a low Poisson’s ratio^6^. Thus all compositions of Ti_3_(Al_1−*x*_Si_*x*_)C_2_ are stable against shear. The interatomic forces in crystalline solids can be predicted as central forces if their Poisson’s ratios range from 0.25 to 0.50, otherwise they will be non-central forces^6^. Clearly, all compositions of Ti_3_(Al_1−*x*_Si_*x*_)C_2_ are stabilized with non-central forces. The non-central force is the characteristic of brittle materials. The nature of chemical bonding is found to be purely covalent or totally metallic for a crystal whose Poisson’s ratio is 0.1 or 0.33^6^. All compositions of Ti_3_(Al_1−*x*_Si_*x*_)C_2_ possess the values between these two characteristic values and exhibit partially metallic and covalent nature. Brittle materials are characterized by a Poisson’s ratio lower than a typical value of 0.26 and ductile materials possess a value larger than 0.26^6^. In this scale, all compositions of Ti_3_(Al_1−*x*_Si_*x*_)C_2_ should exhibit brittleness as predicted from the Cauchy pressure and Pugh’s ratio (see Fig. 2d).

### Thermal properties

#### Debye temperature and melting point

The Debye temperature, a characteristic temperature, can be used to assess many physical properties of solids, including thermal expansion, thermal conductivity, lattice vibrations, melting temperature, and specific heat. It is also linked to the superconducting transition temperature and the electron–phonon coupling constant in the case of superconductors. Moreover, the vacancy formation energy in metals can depend upon the Debye temperature. For calculating this characteristic temperature, the Anderson method is simple and rigorous among several methods. According to this method the Debye temperature *θ*_D_ can be expressed using the average sound velocity *v*_m_ via the expression^42^:

1 $$\theta_{{\text{D}}} = \frac{h}{{k_{{\text{B}}} }}\left[ {\left( {\frac{3n}{{4\pi }}} \right)\frac{{N_{{\text{A}}} \rho }}{M}} \right]^{1/3} v_{{\text{m}}} .$$

Here, *h* and *k*_B_ are respectively the Planck and Boltzmann constants, *N*_A_ is the Avogadro’s number, *ρ* is the density of mass, *n* is the number of atoms in a molecule, and *M* is the molecular weight. The average sound velocity *v*_m_ is calculated from the longitudinal and transverse sound velocities *v*_l_ and *v*_t_ by the equation:

2 $$v_{{\text{m}}} = \left[ {\frac{1}{3}\left( {\frac{1}{{v_{{\text{l}}}^{3} }} + \frac{2}{{v_{{\text{t}}}^{3} }}} \right)} \right]^{ - 1/3} .$$

Using the bulk and shear moduli, *v*_l_ and *v*_t_ can be calculated as

3 $$v_{{\text{l}}} = \left( {\frac{3B + 4G}{{3\rho }}} \right)^{1/2} \;{\text{and}}\;v_{{\text{t}}} = \left( {\frac{G}{\rho }} \right)^{1/2} .$$

**Figure 3:**
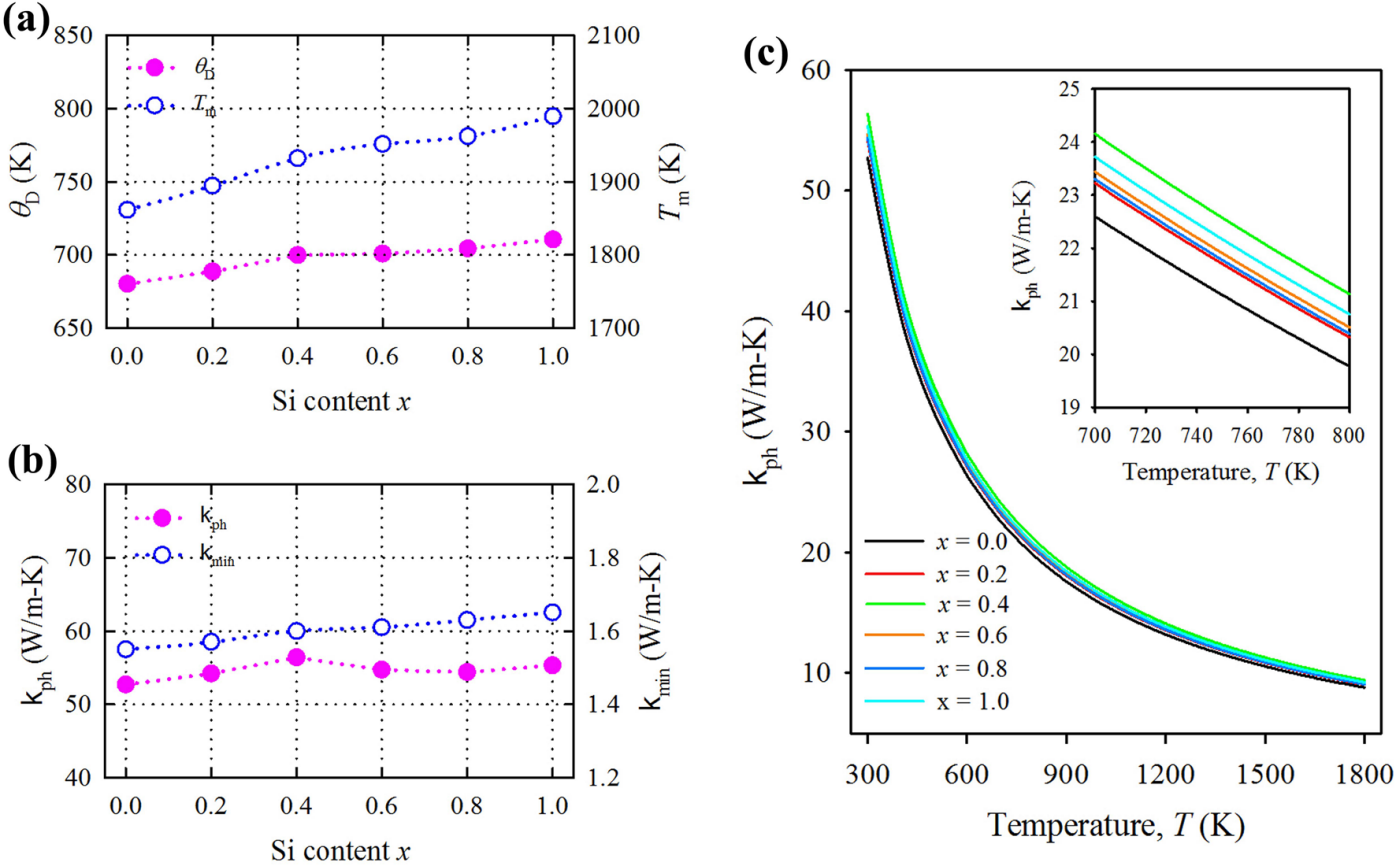
Variation of the investigated thermal properties as a function of Si-content *x* in Ti_3_(Al_1−*x*_Si_*x*_)C_2_ solid solutions. (**a**) Debye and melting temperatures. (**b**) Minimum and room temperature lattice thermal conductivity. (**c**) Temperature dependence of lattice thermal conductivity.

The Debye temperature and sound velocities calculated for Ti_3_(Al_1−*x*_Si_*x*_)C_2_ MAX phases are listed in Table 2 and plotted in Fig. 3a, indicating that the Debye temperature increases with the increase of Si content *x*. High mass density of Si leads to high Debye temperature of Ti_3_(Al_1−*x*_Si_*x*_)C_2_ with the increase of Si content *x*. Commonly, the lower the Debye temperature, the softer the material. Therefore, Ti_3_AlC_2_ is softer than Ti_3_SiC_2_. A comparatively low Debye temperature should lead to low thermal conductivity of Ti_3_AlC, which favors it to be a promising TBC material^43^.

**Table 2:** Density (*ρ*; g/cm^3^), sound velocities (*v*_l_, *v*_*t*_, *v*_m_; km/s), Debye and melting temperatures (*θ*_D_, *T*_m_; K), and minimum and lattice thermal conductivities (*k*_min_, k_ph_; W/m K).

*x* content	*ρ*	*v*_l_	*v*_t_	*v*_m_	*θ*_D_	*T*_m_	*k*_ph_	*k*_min_
0.0	4.2179	8.8497	5.5517	6.1132	680.2	1861.5	52.7	1.55
0.2	4.2837	8.9308	5.5930	6.1599	688.7	1894.5	54.2	1.57
0.4	4.3417	9.0467	5.6582	6.2326	699.9	1932.0	56.4	1.60
0.6	4.3949	9.0590	5.6440	6.2196	700.8	1951.5	54.7	1.61
0.8	4.4461	9.1019	5.6514	6.2300	704.4	1962.0	54.4	1.63
1.0	4.4902	9.1671	5.6827	6.2655	710.5	1989.0	55.3	1.65

An empirical formula: *T*_m_ = 354 + 1.5(2*C*_11_ + *C*_33_) developed for hexagonal crystals is used to calculate the melting point of Ti_3_(Al_1−*x*_Si_*x*_)C_2_ MAX phases^44^. The calculated *T*_m_ is shown in Table 2 and Fig. 3a. It is evident that the melting temperature increases with the increase of Si-content *x*. Higher melting temperature implies that the solid solutions Ti_3_(Al_1−*x*_Si_*x*_)C_2_ should have potential applications in harsh environments.

#### Lattice thermal conductivity

For applications at high temperatures the materials are selected on the basis of their lattice thermal conductivity as well as on their minimum thermal conductivity. The DFT calculation of the lattice thermal conductivity for MAX phases is not simple owing to their dual properties between metals and ceramics. Therefore, a perfect method based on a modest technique with judicious approximations is greatly anticipated. Slack developed an equation to calculate the lattice thermal conductivity taking into consideration the average of the atoms in a “molecule” (or the atoms in the formula unit of the crystal) and their average atomic weight^45^. Slack’s model is convenient to determine the temperature-dependent lattice thermal conductivity of MAX phases owing to their partially ceramic nature^46^, while Clarke’s model is very expedient to calculate the temperature-independent minimum thermal conductivity of compounds. The empirical formula derived by Slack for calculating the lattice thermal conductivity is

4 $$k_{{{\text{ph}}}} = A\frac{{M_{{{\text{av}}}} \theta_{D}^{3} \delta }}{{\gamma^{2} n^{2/3} T}}.$$

In this equation, *M*_av_ denotes the average atomic mass of a crystal in kg/mol, *θ*_D_ refers to the Debye temperature in K, *δ* defines the cubic root of average atomic volume in m, *n* is the number of atoms in the conventional unit cell, *T* is the temperature in K, and γ is the unitless Grüneisen parameter, which is derived from the Poisson’s ratio using the expression:

5 $$\gamma = \frac{{3\left( {1 + \nu } \right)}}{{2\left( {2 - 3\nu } \right)}}.$$

The factor *A*(*γ*) due to Julian^47^ is calculated as

6 $$A\left( \gamma \right) = \frac{{5.720 \times 10^{7} \times 0.849}}{{2 \times \left( {1 - 0.514/\gamma + 0.228/\gamma^{2} } \right)}}.$$

The lattice thermal conductivity calculated at room temperature (300 K) for Ti_3_(Al_1−*x*_Si_*x*_)C_2_ solid solutions is listed in Table 2 and their room temperature values and temperature dependencies are shown in Fig. 3b,c, respectively. The reliability of the Slack model has been established as evident from the calculated lattice thermal conductivity of Ta_4_AlC_3_ (5 W/m–K) being comparable with the experimental value of 6 W/m–K at 1300 K. Moreover, the calculated value for Nb_4_AlC_3_ is identical to the experimental value of 7 W/m–K at 1300 K^48^. With Si-content *x* the lattice thermal conductivity of Ti_3_(Al_1−*x*_Si_*x*_)C_2_ decreases gradually.

The total thermal conductivity at room temperature for MAX phases ranges from 12 to 60 W/m–K in which the electronic contribution to the total thermal conductivity is trivial^49^. Therefore, it is anticipated that the present values for Ti_3_(Al_1−*x*_Si_*x*_)C_2_ solid solutions may not exceed this range if their electronic contribution is considered. The lattice thermal conductivity of Ti_3_(Al_1−*x*_Si_*x*_)C_2_ solid solutions at room temperature ranges from 52.7 to 55.3 W/m–K. From Fig. 3c, it is evident that the lattice thermal conductivity decreases gradually with increasing temperature. The compositions with *x* = 0.0 and *x* = 0.4, respectively have the lowest and highest lattice thermal conductivity in the whole temperature range. The lattice thermal conductivity of Ti_3_(Al_1−*x*_Si_*x*_)C_2_ solid solutions follows the order with *x*: 0.4 > 1.0 > 0.6 > 0.8 > 0.2 > 0.0.

#### Minimum thermal conductivity

At high temperatures, the inherent thermal conductivity reaches a lower limit, which is known as the minimum thermal conductivity of the compound. At the high temperature, the phonons become completely unpaired and the heat energy transfers to neighboring atoms. In this situation, the mean free path of the phonons is supposed to be the average interatomic distance. Thus, different atoms in a molecule can be replaced with an equivalent atom that has an average atomic mass of *M*/*n* (*n* is the number of atoms in the unit cell) in this approximation. In the cell, a single “equivalent atom” never has the optical modes and can be applied to derive a formula for the minimum thermal conductivity *κ*_min_ at high temperature as assumed in Clarke’s model^50^:

7 $$\kappa_{\min } = { }k_{{\text{B}}} v_{{\text{m}}} \left( {\frac{{nN_{{\text{A}}} \rho }}{M}} \right)^{2/3} .$$

The symbols in this equation bear the same meanings as in Eq. (4). The minimum thermal conductivity calculated for Ti_3_(Al_1−*x*_Si_*x*_)C_2_ solid solutions is shown in Table 2 and Fig. 3b. The minimum thermal conductivity increases with the increase of Si-content *x*. The minimum thermal conductivity shows linear increasing trend with the Si content *x*. The end member Ti_3_AlC_2_ has a minimum thermal conductivity smaller than that of other compositions including the other end member Ti_3_SiC_2_ too.

### Optical properties

The optical properties of Ti_3_(Al_1−*x*_Si_*x*_)C_2_ MAX phase solid solutions are calculated for the two polarization directions 〈100〉 and 〈001〉 of incident photons. Due to the hexagonal symmetry of MAX phases the incident photon polarization directions 〈100〉 and 〈001〉 correspond to the directions of associated electric field perpendicular and parallel to the crystallographic c-axis, respectively. MAX phases are partially metallic compounds and consequently the intraband transitions have momentous impact at far infrared regions i.e., low energy part of the spectrum. To take account of intraband transition, a phenomenological damping of 0.05 eV, Drude parameters, i.e., the free-electron plasma frequency of 3 eV and Gaussian smearing of 0.5 eV are used for all calculations. The imaginary part of the dielectric function that leads to calculate the remaining optical properties of materials can be expressed as:

8 $$\varepsilon_{2} \left( \omega \right) = \frac{{2\pi e^{2} }}{{{\Omega }\varepsilon_{0} }}\mathop \sum \limits_{k,v,c} \left| {\psi_{k}^{c} } \right|{\varvec{u}}.{\varvec{r}}\left| {\psi_{k}^{v} } \right|^{2} \delta \left( {E_{k}^{c} - E_{k}^{v} - E} \right)$$

Here, *ω* is the frequency of the phonon, *e* is the electronic charge, Ω is the unit cell volume, **u** is the unit vector along the polarization of the incident electric field and $\psi_{k}^{c}$ and $\psi_{k}^{v}$ are wave functions for conduction and valence band electrons at a particular *k*, respectively. The expressions for the remaining functions can be found in the literature^51^.

**Figure 4:**
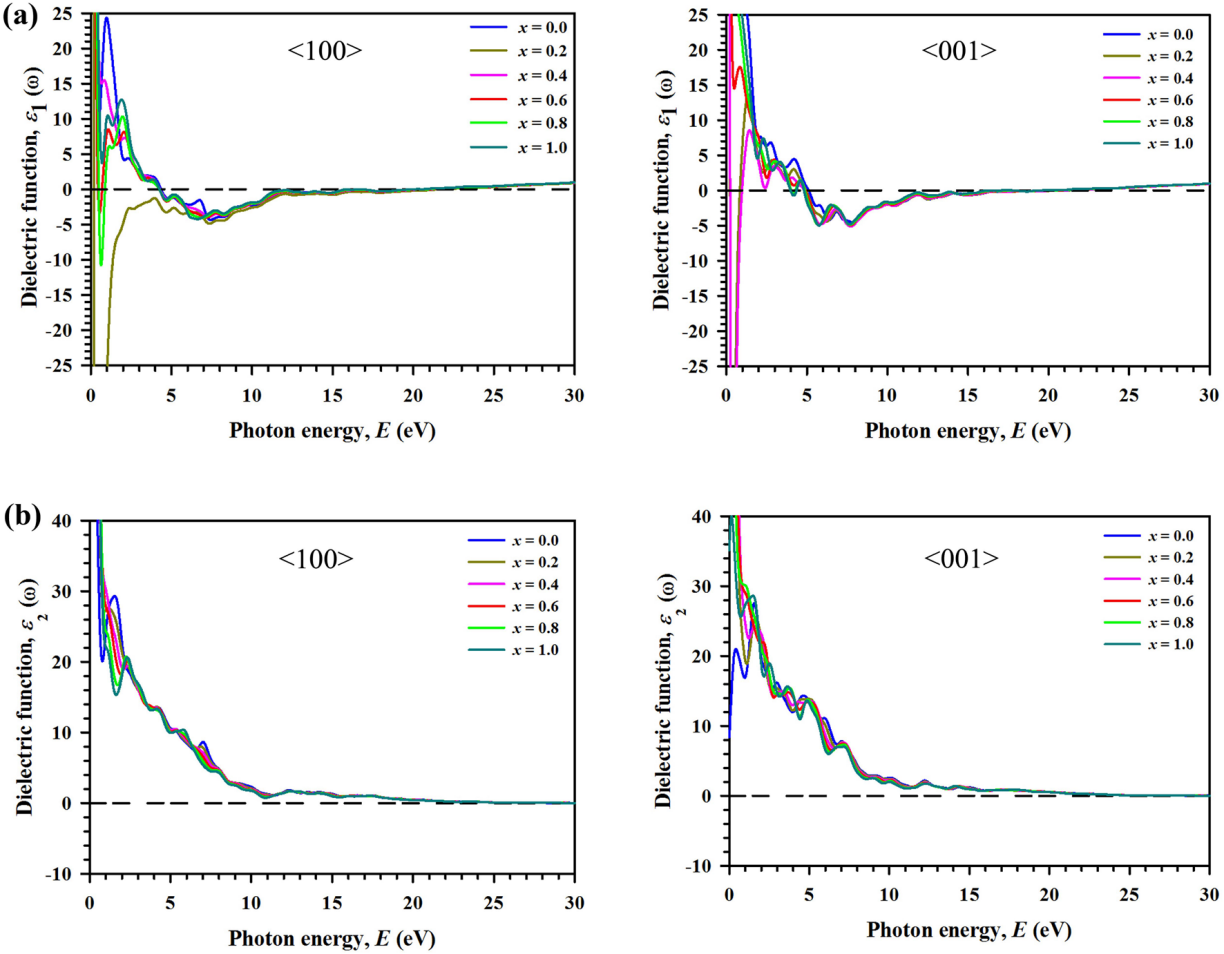
Variation of dielectric function as a function of incident photon energy ranged from 0 to 30 eV along the polarization directions 〈100〉 and 〈001〉 for the considered solid solutions Ti_3_(Al_1−*x*_Si_*x*_)C_2_. (**a**) Real part of dielectric function *ε*_1_(ω). (**b**) Imaginary part of dielectric function *ε*_2_(ω).

The real part of the dielectric function *ε*_1_(ω) calculated for photon energy of 30 eV is shown in Fig. 4a. Comparing the spectra for 〈100〉 and 〈001〉 polarization directions from left and right panels of Fig. 4a, it can be said that the solid solutions exhibit highly anisotropic nature at the low energy region from 0 to ~ 6 eV. It is seen that the real part *ε*_1_(ω) goes through zero in the low energy range, indicating the metallic nature for all compositions of Ti_3_(Al_1−*x*_Si_*x*_)C_2_. The static dielectric constant is higher for composition *x* = 0.2 and lowest for *x* = 1 when photon is polarized along the 〈100〉 direction. For polarization direction 〈001〉, the static dielectric function is highest for *x* = 0.4 and lowest for *x* = 1. All spectra approach zero at 15.5 eV for polarization direction 〈100〉 and at 16 eV for polarization direction 〈001〉. The dielectric function shows highest anisotropic nature for *x* = 0.4 and lowest for *x* = 1.

Regarding optical phenomena, the imaginary part of the dielectric function *ε*_2_(*ω*) reveals the energy attenuation features of an optical medium with the change of frequency. Figure 4b shows the spectra of *ε*_2_(*ω*) calculated for Ti_3_(Al_1−*x*_Si_*x*_)C_2_ MAX solid solutions. For both polarization directions it approaches zero from above, indicating the metallic nature of all compositions. At the IR and visible light regions, the spectral features are different for the two polarization directions, highlighting the anisotropic nature in optical properties. At the high energy region the spectra for both polarizations are almost identical for all compositions. The electronic structure of a material is mostly accountable for optical spectra. For this reason, the origin of the peaks in the spectra can be explained from the DOS plot of the relevant material. To elucidate this link, the composition *x* = 0 is selected arbitrarily. In the spectrum for polarization 〈001〉, the peak nearby 0.4 eV is due to transitions within Ti 3d orbitals.

**Figure 5:**
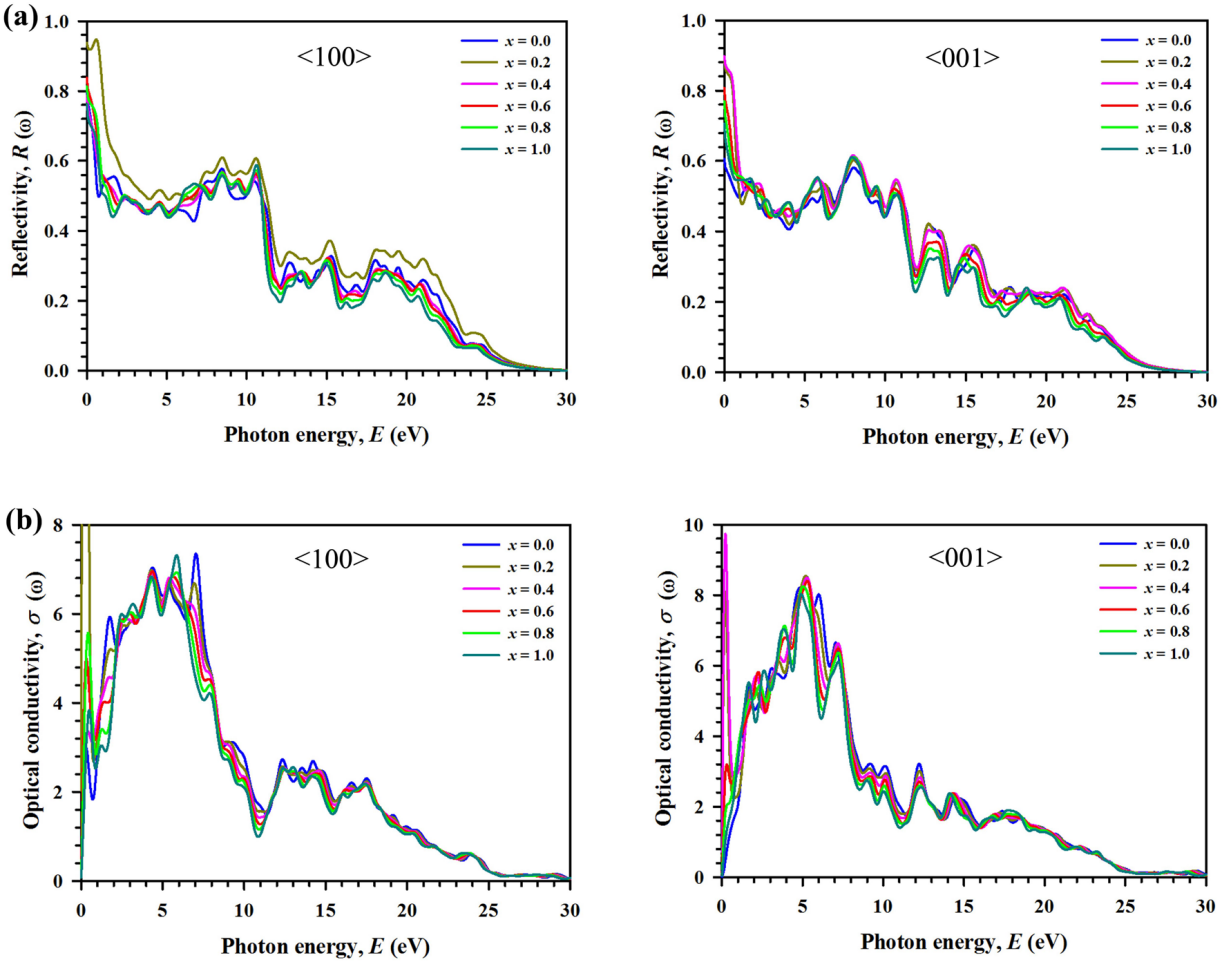
Variation of optical properties as a function of incident photon energy ranged from 0 to 30 eV along the polarization directions 〈100〉 and 〈001〉 for the considered solid solutions Ti_3_(Al_1–*x*_Si_*x*_)C_2_. (**a**) Reflectivity *R*(ω). (**b**) Optical conductivity σ(ω).

The reflectivity of Ti_3_(Al_1−*x*_Si_*x*_)C_2_ MAX phases is calculated for the 〈100〉 and 〈001〉 polarization directions and is shown in Fig. 5a. The spectra for both polarizations exhibit distinct features for all compositions with peak’s shapes, heights and positions, indicating anisotropic nature. In the visible region (1.8–3.1 eV), the minimum reflectivity of all compositions for both polarizations is above 45%, which makes them potential candidate materials for coatings to reduce solar heating^52^. With reflectivity above 51%, the composition with *x* = 0.2 is a better coating material for preventing solar heating than the other compositions. In the UV region, all compositions exhibit maximum reflectivity between 7.3 and 10.8 eV for the polarization direction 〈100〉. The reflectivity spectra start to decrease drastically at around 21 eV and reach zero value at around 30 eV for both polarizations.

The optical conductivity *σ*(ω) is a key parameter for understanding the electromagnetic response of a material. In the presence of an alternating electric field, it implies electrical conductivity of the material. The photoconductivity can be estimated by means of optical conductivity^51^. The optical conductivity of Ti_3_(Al_1−*x*_Si_*x*_)C_2_ is calculated for the 〈100〉 and 〈001〉 polarization directions, and its real parts are shown in Fig. 5b. The spectra of *σ*(ω) for both polarization directions show significant diversity in a wide range of photon energy of 0 to 25 eV for all compositions, indicating the anisotropic nature of the optical properties. In the higher-energy region from 25 to 30 eV, the features of the optical conductivity are almost identical for both polarization directions. The peaks in the spectra arise mainly from interband transitions from occupied Ti p orbitals to unoccupied Ti d orbitals.

We further calculate other optical properties such as refractive index, extinction coefficient, absorption coefficient and energy loss function and the results are presented in Supplementary Information section (see Supplementary Figs. 3 & 4).

## Conclusion

In summary, we have employed DFT for Ti_3_(Al_1−*x*_Si_*x*_)C_2_ solid solutions to calculate the physical properties including structural, electronic, mechanical, thermal and optical properties for the first time. The lattice parameters and elastic moduli calculated in this study show reasonable agreement with experimental results. With the increase of Si content *x*, a decrease of cell parameters (negligible in *a* and significant in *c* as well as in *V*) occurs. All elastic constants and moduli of Ti_3_(Al_1−*x*_Si_*x*_)C_2_ are found to be increased by the Si content *x*. The Fermi level gradually shifts to the upper bound of the pseudogap when the Si content increases from zero to unity, signifying a gradual decrease in structural stability of the Ti_3_(Al_1−*x*_Si_x_)C_2_ system as *x* increases. All Ti_3_(Al_1−*x*_Si_*x*_)C_2_ compositions are brittle in nature in accordance with the Cauchy pressure, Pugh’s ratio and Poisson’s ratio. The Si content *x* increases the stiffness and decreases the thermal shock resistance of Ti_3_(Al_1−*x*_Si_*x*_)C_2_. Ti_3_AlC_2_ is expected to be a promising thermal barrier coating material due to its low Debye temperature, lattice thermal conductivity and minimum thermal conductivity. Due to high melting temperature the solid solutions Ti_3_(Al_1−*x*_Si_*x*_)C_2_ should also have potential applications in harsh environments. All compositions of Ti_3_(Al_1−*x*_Si_*x*_)C_2_ are expected to be potential candidate coating materials for reduction of solar heating due to their minimum reflectivity above 45% in the visible region (1.8–3.1 eV).

### Computational methods

Electronic structure calculations are carried out using the pseudopotential DFT method embodied in the Cambridge serial total energy package (CASTEP) code^53^. The Perdew-Burke-Ernzerhof (PBE) generalized gradient approximation (GGA) is employed to model the exchange–correlation potential^54^. The interaction between electrons and ion cores is treated in the reciprocal space using the Vanderbilt-type ultrasoft pseudopotential^55^. For the Brillouin zone sampling a special k-point mesh of 13 × 13 × 2 in Monkhorst–Pack scheme is used^56^. BFGS minimization technique named after Broyden–Fletcher–Goldferb–Shanno is applied to optimize the geometry via minimizing the total energy and internal forces^57^. Pseudoatomic calculations for the valence and nearly valence electrons of Ti, Al, Si and C are performed using a planewave basis with an energy cutoff of 500 eV. The convergence tolerance for the total energy, maximum ionic force, maximum ionic displacement and maximum stress are set to 5.0 × 10^–6^ eV/atom, 0.01 eV/Å, 5.0 × 10^–4^ Å and 0.02 GPa, respectively. The present DFT method has been established for calculating the electronic structure of crystalline solids as described in previous studies^3, 5^.

The conventional unit cell of Ti_3_(Al_1−x_Si_x_)C_2_ is modeled with the Virtual Crystal Approximation (VCA) technique as described in the code for mixing Si with Al at A-atomic sites, and the lattice parameters are obtained from the optimized cells. This technique allows one to mix different atoms, isotopes and oxidation states for a specific atomic site to be occupied randomly. The VCA technique has been previously applied successfully to study the disorder in some perovskites, silicates, ferroelectric ceramics and MAX phases^18,19,24^. This approach does not allow for any probable short-range order. It is assumed that a virtual atom occupies every potentially disordered site and extrapolates an averaged behavior between the real components. The VCA method ignores local distortions nearby the atoms. Therefore, in our case, there may be some energy difference between the actual Al_1−*x*_Si_*x*_ model and the VCA scheme. Ramer and Rappe^58^ carried out a comparative study for three different modifications of the 1:1 Pb(Zr,Ti)O_3_ ceramics. They found that the VCA results for structural aspect are highly consistent with those obtained in calculations. For total energies, both sets of results exhibit a negligibly small difference.

For optical properties and Mulliken atomic population calculations, a 5 × 1 × 1 supercell of Ti_3_AlC_2_ is constructed, from which 2, 4, 6, 8, and 10 Al atoms are replaced by Si atoms successively to obtain the composition Ti_3_(Al_1−x_Si_x_)C_2_ with x = 0.2, 0.4, 0.6, 0.8, and 1.0, respectively. In this calculation, a k-point mesh of 2 × 9 × 1-grid with a plane-wave energy cutoff of 350 eV is applied.

## Supplementary Information

Supplementary Information 1.
