# Supplementary material for: Effects of Al substitution by Si in Ti3AlC2 nanolaminate
Source: Sci Rep. 2021 Feb 9;11:3410. doi: 10.1038/s41598-021-81346-w (PMC7873089; doi:10.1038/s41598-021-81346-w)
Supplement: Supplementary file 1 — Supplementary Information 1. [file 41598_2021_81346_MOESM1_ESM.docx]

Supplementary Information:

Effects of Al substitution by Si in Ti_3_AlC_2_ nanolaminate

**M. A. Hadi^1,*^ , Md Roknuzzaman^2,3,4^, M. T. Nasir^5^, U. Monira^1^, S. H. Naqib^1^,** **A. Chroneos^6,7^, A.K.M. A. Islam^1,8^_,_ Jose A. Alarco^2^, Kostya (Ken) Ostrikov^2^**

**^*^Correspondence: hadipab@gmail.com (M.A. Hadi)**

*^1^Department of Physics, University of Rajshahi, Rajshahi 6205, Bangladesh*

*^2^School of Chemistry and Physics and Centre for Materials Science, Queensland University of Technology (QUT), Brisbane, QLD 4000, Australia*

*^3^School of Physics, University of New South Wales (UNSW Sydney), Kensington, Sydney, NSW 2052, Australia*

*^4^Department of Physics, Jashore University of Science and Technology, Jashore 7408, Bangladesh*

*^5^Department of Arts & Sciences, Bangladesh Army University of Science and Technology, Saidpur 5310, Nilphamari, Bangladesh*

*^6^Faculty of Engineering, Environment and Computing, Coventry University, Priory Street, Coventry CV1 5FB, UK*

*^7^Department of Materials, Imperial College, London SW7 2AZ, UK*

*^8^International Islamic University Chittagong, Kumira, Chittagong 4318, Bangladesh*

**Supplementary Table** 1. Structural properties of Ti_3_(Al_1-_*_x_*Si*_x_*)C_2_ solid solutions

| Si content *x* | *a* (Å) | *c* (Å) | *c*/*a* | *z*_C_ | *V* (Å^3^) | Remarks |
| --- | --- | --- | --- | --- | --- | --- |
| 0.0 | 3.0783 | 18.6693 | 6.0724 | 0.069289 | 153.21 | VCA (This Calc.) |
|  | 3.0730 | 18.5410 | 6.0335 |  | 151.63 | Experimental^1^ |
| 0.2 | 3.0752 | 18.4409 | 5.9967 | 0.070039 | 151.03 | VCA (This Calc.) |
|  | 3.0717 | 18.2889 | 5.9540 |  | 149.44 | Experimental^1^ |
| 0.4 | 3.0727 | 18.2451 | 5.9378 | 0.070680 | 149.18 | VCA (This Calc.) |
|  | 3.0704 | 18.0809 | 5.8884 |  | 147.62 | Experimental^1^ |
| 0.6 | 3.0706 | 18.0683 | 5.8844 | 0.071218 | 147.53 | VCA (This Calc.) |
|  | 3.0698 | 17.8761 | 5.8232 |  | 145.89 | Experimental^1^ |
| 0.8 | 3.0702 | 17.8864 | 5.8258 | 0.071770 | 146.01 | VCA (This Calc.) |
|  | 3.0679 | 17.7248 | 5.7775 |  | 144.48 | Experimental^1^ |
| 1.0 | 3.0720 | 17.7097 | 5.7649 | 0.072356 | 144.74 | VCA (This Calc.) |
|  | 3.0660 | 17.6681 | 5.7626 |  | 143.84 | Experimental^1^ |


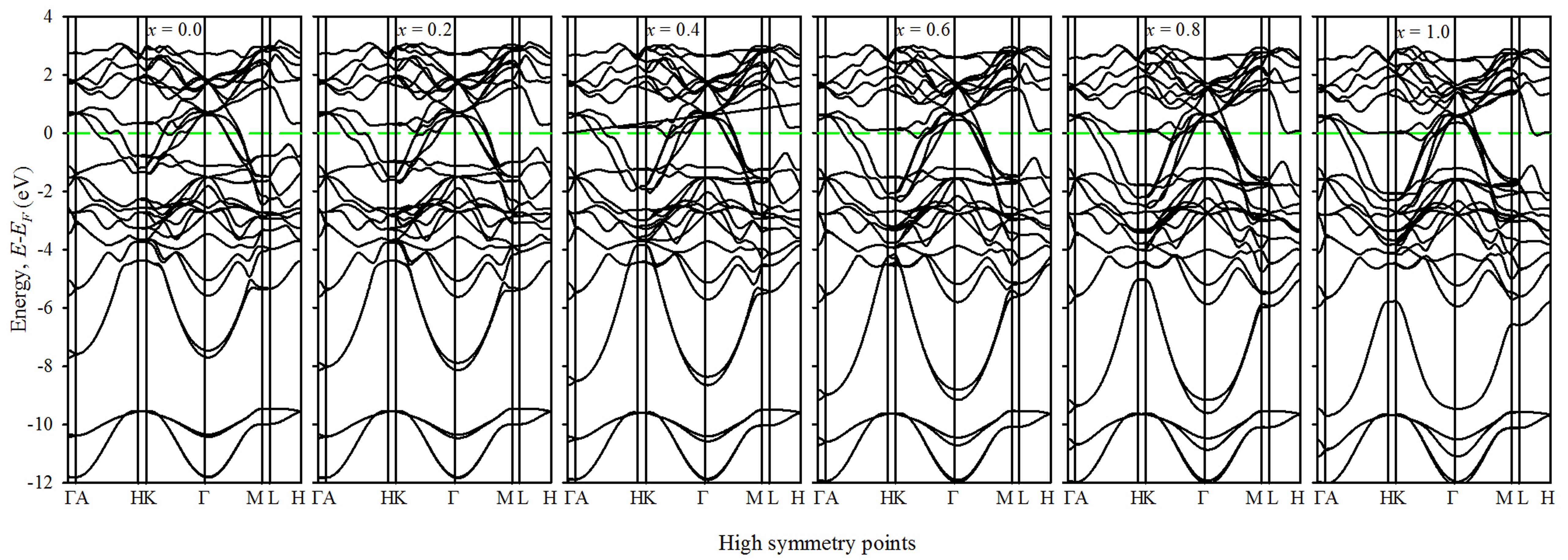


**Supplementary Figure 1.** Electronic band structures of Ti_3_(Al_1-_*_x_*Si*_x_*)C_2_ MAX phases.


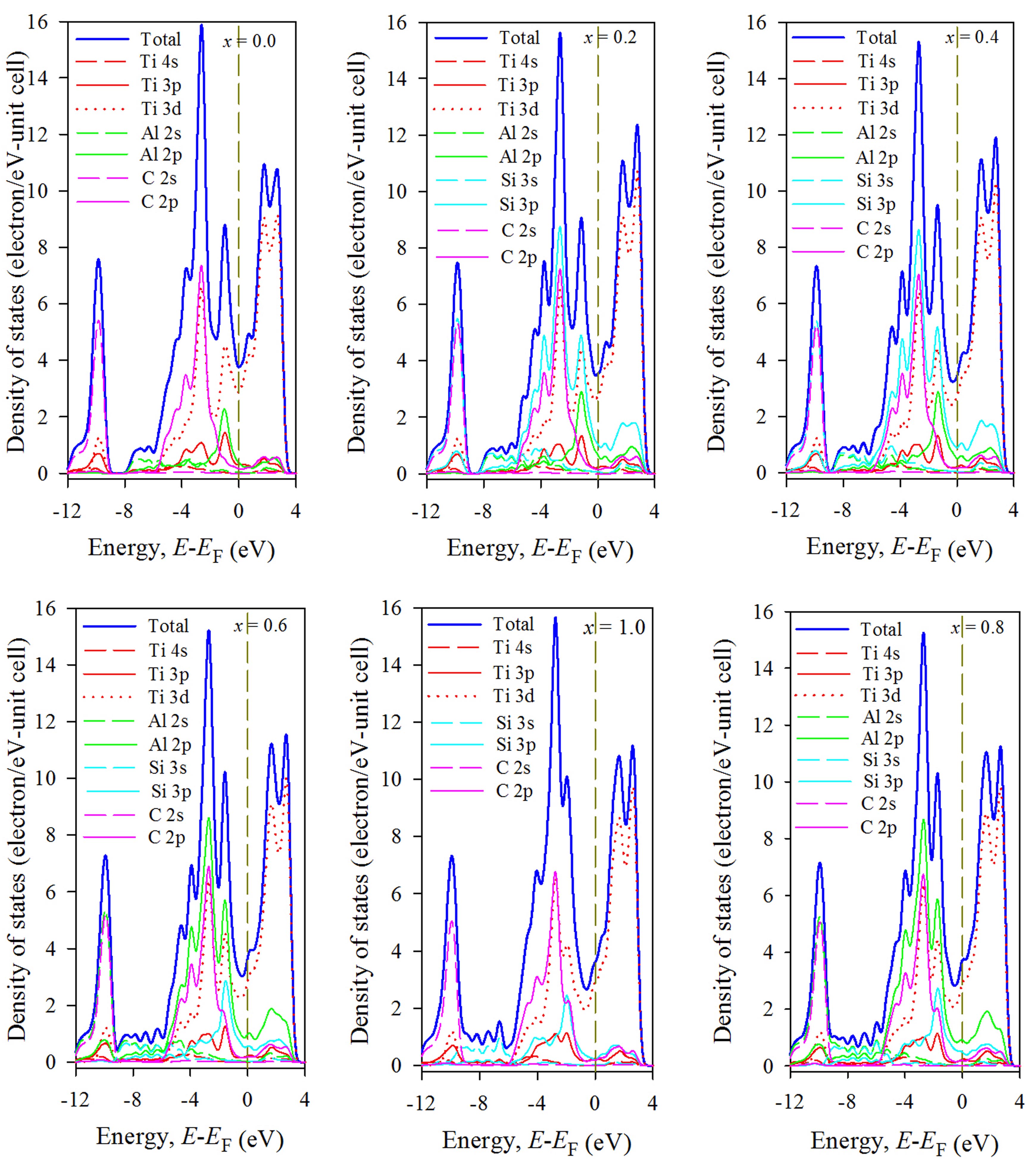


**Supplementary Figure 2.** Electronic density of states (DOS) of Ti_3_(Al_1-_*_x_*Si*_x_*)C_2_ as a function of Si content *x*.


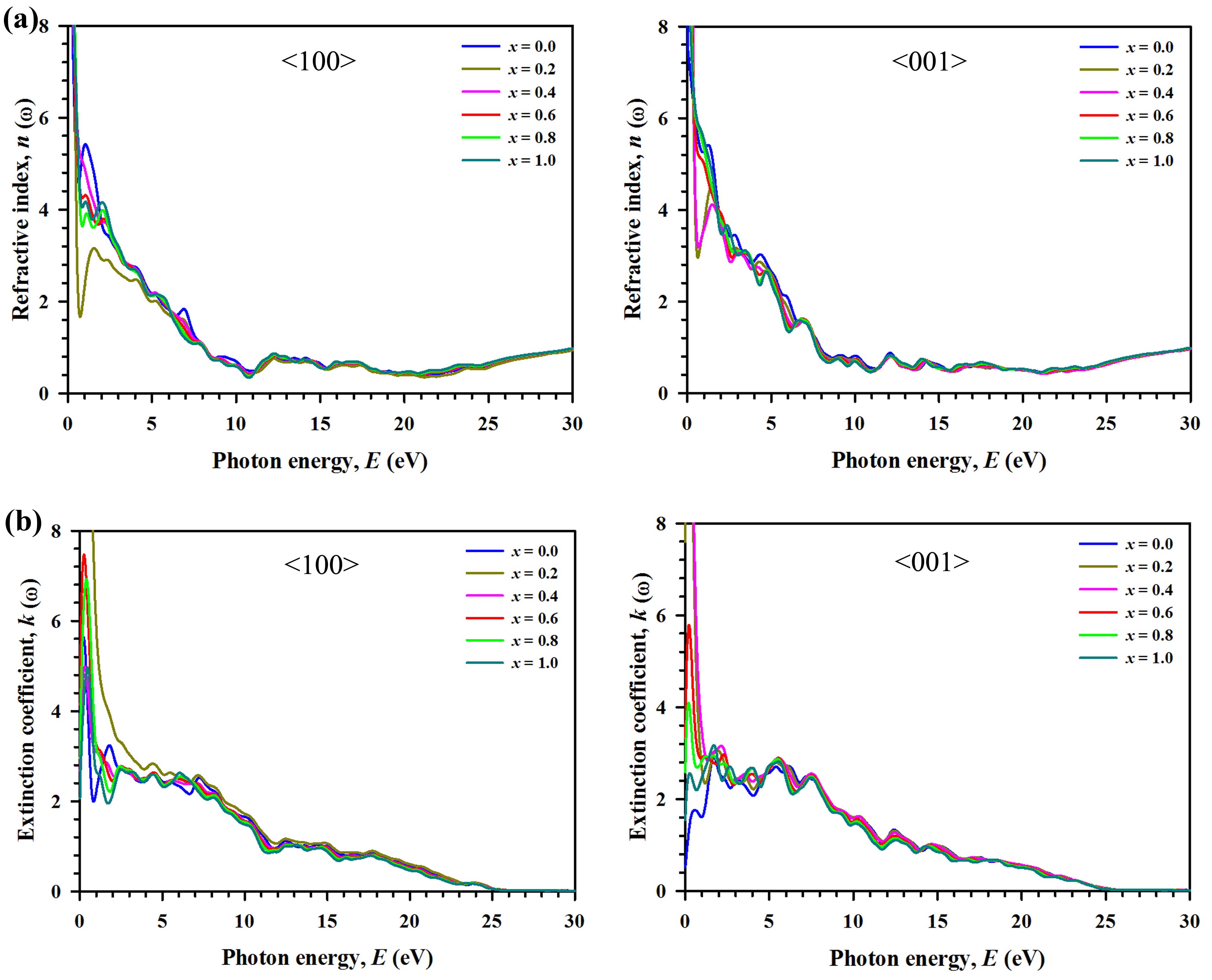


**Supplementary Figure 3.** (a) Refractive index *n*(ω) and (b) Extinction coefficient *k*(ω)for 〈100〉 and 〈001〉 polarization.

**Refractive index.** The exact knowledge of the refractive index *n*(*ω*) of a material serves as a guide for perfect design of electronic devices. The refractive index of all compositions of Ti_3_(Al_1-_*_x_*Si*_x_*)C_2_ is calculated for 〈100〉 and 〈001〉 polarization directions and is plotted in Supplementary Figure 3(a). For polarization 〈100〉 the static value of the refractive index *n*(0) is highest for *x* = 0.2 and lowest for *x* = 1, while for polarization 〈001〉 the value of *n*(0) is highest for *x* = 0.4 and lowest for *x* = 0. The peaks in each spectrum in the moderate IR region are due to the intraband transitions of electrons. The *n*(*ω*) spectra show a rapid decrease starting at 0.45 eV and reach a lowest value of 0.44 at around 10.8 eV. The spectra show anisotropic character up to 20eV and then exhibit almost similar features up to 30 eV for all compositions.

**Extinction coefficient.** The extinction coefficient *k*(*ω*), imaginary part of the complex index of refraction, can serve as an additional key optical parameter. It refers to the attenuation of electromagnetic radiation in a medium and measures how strongly a material absorbs radiation incident upon it at a particular wavelength per mass density or per molar concentration. The spectra of *k*(*ω*) calculated for the 〈100〉 and 〈001〉 polarizations are shown in Supplementary Figure 3(b). It is linked to the conductive properties of the material. Metallic materials have a large static extinction coefficient, whereas semiconductor materials have a small one. On the other hand, dielectric materials are basically nonconductors whose static extinction coefficient is zero. A large extinction coefficient at low photon energy indicates metallic conductivity of all compositions of Ti_3_(Al_1-_*_x_*Si*_x_*)C_2_. Similar to *n*(0), for polarization 〈100〉 the static value of the extinction coefficient *k*(0) is highest for *x* = 0.2 and lowest for *x* = 1, while for polarization 〈001〉 the value of *k*(0) is highest for *x* = 0.4 and lowest for *x* = 0.


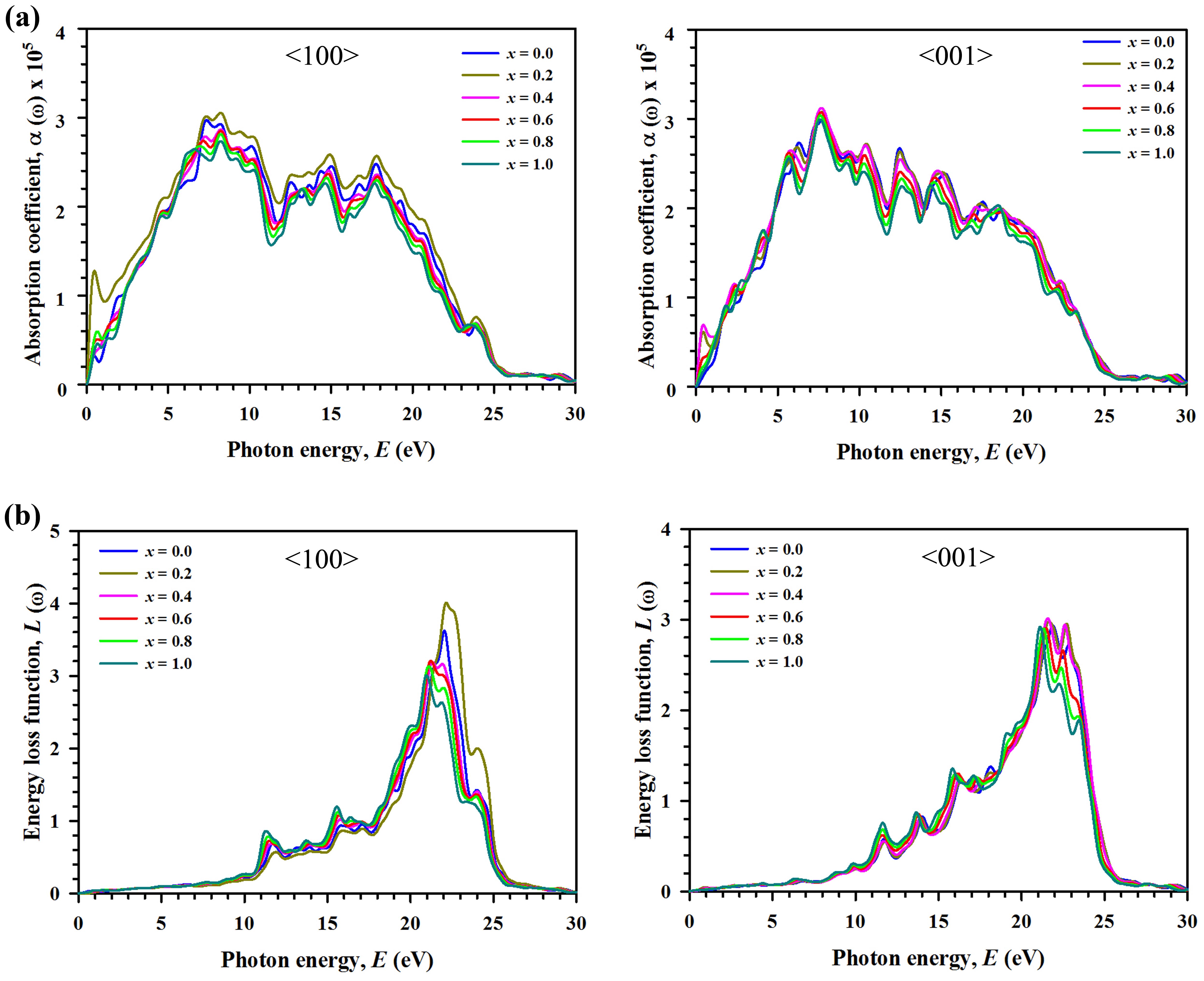


**Supplementary Figure 4.** (a) Absorption coefficient *α*(ω) and (b) Energy loss function L(ω)for 〈100〉 and 〈001〉 polarization.

**Absorption coefficient.** The absorption coefficient *α*(*ω*) is a measure of the penetration of light at a particular wavelength into a material before being absorbed. It also provides information regarding the optimum solar energy conversion efficiency, which is important for the practical application of a material in solar cells. The spectra of *α*(*ω*) calculated for polarization directions 〈100〉 and 〈001〉 are shown in Supplementary Figure 4(a). Absorption starts at zero photon energy, indicating the metallic nature of all compositions. Each spectra show their highest peak at around 8.2 eV for polarization 〈100〉 and at around 7.7 eV for polarization 〈001〉. The spectral features for both polarizations are distinctly different. Highest absorption occurs for the composition with *x* = 0.2 for polarization 〈100〉 and for polarization 〈001〉 the highest absorption occurs for the composition with *x* = 0.4. For both polarizations, the lowest absorption occurs for the composition with *x* = 1. There is no significant change in the energy range between 25 and 30 eV for both polarizations. It is notable that the static absorption coefficient *α*(0) exhibits a universal nonzero value for all compositions of Ti_3_(Al_1-_*_x_*Si*_x_*)C_2_ MAX phases like other hexagonal systems^2^.

**Energy loss function.** The energy loss function *L*(*ω*) refers to the energy loss of the first electron passing through a material. The calculated energy loss functions of Ti_3_(Al_1-_*_x_*Si*_x_*)C_2_ MAX phases for the 〈100〉 and 〈001〉 polarization directions are shown in Supplementary Figure 4(b). The energy loss spectrum represents the frequency of collective oscillations of the valence electrons, and its peak refers to the nature of plasma oscillation and is related to a distinctive frequency well-known as the plasma frequency *ω*_p_ of the material. At *ω*_p_, the real part of the dielectric function, *ε*_1_(*ω*), changes from negative to positive together with the imaginary part of the dielectric function, *ε*_2_(*ω*) < 1. At the plasma frequency, a material changes from metallic to dielectric response. From the energy loss spectra, the plasma frequencies of Ti_3_(Al_1-_*_x_*Si*_x_*)C_2_ MAX phases are within 20.9 – 22.2 eV for 〈100〉 polarization and 21.1 – 22.8 eV for 〈001〉 polarization and it is also consistent with the studied real part of the dielectric function as shown in Figure 4(a). The plasma frequency is highest for the composition with *x* = 0.2 for both polarizations. There are no peaks in the energy loss spectra within the energy range from 0 to 10 eV as a consequence of the large *ε*_2_(*ω*)^3^ as shown in Figure 4(b).

**References**

1. Gao, H., Beniteza, R., Son, W., Arroyave, R. & Radovic M. Structural, physical and mechanical properties of Ti_3_(Al_1−x_Si_x_)C_2_ solid solution with x=0–1. *Mater. Sci. Engineer. A* **676**, 197–208 (2016).

2. Dhar, N. & Jana, D. Magnetic and optical properties of carbon and silicon decorated freestanding buckled germanene: A DFT approach. *J. Phys. Chem. Solids* **115**, 332–341 (2018).

3. Roknuzzaman, [M.](https://www.sciencedirect.com/science/article/abs/pii/S0925838817328803#!) *et al.* First hafnium-based MAX phase in the 312 family, Hf_3_AlC_2_: A first-principles study. *J. Alloys Comp.* **727**, 616-626 (2017).
